# Correlations between Retinal Microvascular Parameters and Clinical Parameters in Young Patients with Type 1 Diabetes Mellitus: An Optical Coherence Tomography Angiography Study

**DOI:** 10.3390/diagnostics14030317

**Published:** 2024-02-01

**Authors:** Alexandra Oltea Dan, Carmen Luminița Mocanu, Andrei Teodor Bălășoiu, Cornelia Andreea Tănasie, Ileana Puiu, Anca Elena Târtea, Veronica Sfredel

**Affiliations:** 1 Department of Physiology, University of Medicine and Pharmacy of Craiova, 200349 Craiova, Romania; puiu.alexandra.oltea@gmail.com (A.O.D.);; 2 Department of Ophthalmology, University of Medicine and Pharmacy of Craiova, 200349 Craiova, Romania; 3 Department of Pediatrics, University of Medicine and Pharmacy of Craiova, 200349 Craiova, Romania; ileana.puiu@umfcv.ro; 4 Department of Neurology, University of Medicine and Pharmacy of Craiova, 200349 Craiova, Romania

**Keywords:** optical coherence tomography angiography, non-invasive retinal imaging, biomarkers, type 1 diabetes mellitus, diabetic retinopathy

## Abstract

Objectives: In the current study, we investigated the correlations between retinal microvascular parameters using optical coherence tomography angiography (OCTA) and clinical parameters for a group of 69 young patients with type 1 diabetes mellitus (T1DM). Materials and Methods: This retrospective, exploratory study enrolled 69 patients between 5 years old and 30 years old who met the inclusion criteria. All the study participants underwent a comprehensive ophthalmic examination and OCTA scans for the evaluation of the retinal microcirculation. The retinal OCTA parameters were correlated with the following clinical parameters: the patient’s age at the onset of the disease, the duration of T1DM, the BMI at the time of enrollment in the study, the HbA1C values at onset, the mean values of HbA1C over the period of monitoring the disease and the degree of DKA at onset. Results: For the study group, the foveal avascular zone (FAZ) area and perimeter correlated positively with the mean value of HbA1C (Pearson correlation, Sig.2-Tailed Area: 0.044; perimeter: 0.049). The total vessel density in the superficial capillary plexus (SCP) correlated negatively with the duration of T1DM, based on the superior and inferior analyzed areas (Spearman correlation, Sig.2-Tailed SCP in total region: 0.002; SCP in the superior region: 0.024; SCP in the inferior region: 0.050). The foveal thickness also correlated negatively with the levels of diabetic ketoacidosis (DKA) at onset (Spearman correlation, Sig.2-Tailed: 0.034) and the levels of HbA1C at onset (Spearman correlation, Sig.2-Tailed: 0.047). Further on, the study patients were distributed into two groups according to the duration of the disease: group 1 included 32 patients with a duration of T1DM of less than 5 years, and group 2 included 37 patients with a duration of T1DM of more than 5 years. Independent *t*-tests were used to compare the OCTA retinal parameters for the two subgroups. While the FAZ-related parameters did not show significant statistical differences between the two groups, the vessel densities in both the SCP and DCP were significantly lower in group 2. Conclusions: Our data suggest that specific alterations in OCTA imaging biomarkers correlate with various clinical parameters: the FAZ area and perimeter increase with higher mean values of HbA1C, leading to poor metabolic control. Moreover, the SCP total vessel density decreases as the duration of T1DM increases. Regarding the vessel densities in the SCP and the DCP, they decrease with a duration of the disease of more than 5 years.

## 1. Introduction

Diabetes mellitus (DM) is a major chronic health condition, as it has one of the world’s fastest-growing incidences [1]. It is currently affecting approximately 460 million people worldwide, with an estimated increase to approximately 642 million people by 2040 [2], with a subsequent significant increase in both microvascular and macrovascular complications. Therefore, identifying new imaging biomarkers and novel therapeutic targets for improving the management of DM and its complications has become essential. [3].

Type 1 diabetes mellitus (T1DM) represents one of the most common chronic diseases during childhood, with a significantly increasing incidence [4] of approximately 3 new cases per 100,000 people under 18 years old yearly [5]. This metabolic condition occurs through the autoimmune destruction of the pancreatic beta cells and a subsequent severe impairment of insulin production [6]. Insulin administration represents the only effective treatment for patients with T1DM [7]. Although multiple insulin delivery systems, such as daily subcutaneous injections, continuous subcutaneous insulin infusions or insulin pumps, are available, most T1DM patients fail to achieve effective management of their blood glucose levels [8].

According to recent research, children who initially present with severe diabetic ketoacidosis (DKA) at the onset of T1DM have poorer long-term blood sugar management, as indicated by glycated hemoglobin (HbA1C) levels. Consequently, they require more intensive treatment and more frequent monitoring [9].

Diabetic retinopathy (DR) is the most common microvascular complication of T1DM and the leading cause of irreversible visual loss [10] for diabetic patients. The clinical diagnosis of DR also represents a strong predictor of macrovascular dysfunction [11]. In the early stages of DR, hyperglycemia and altered metabolic pathways lead to vascular endothelial damage, with the development of microaneurysms and intraretinal hemorrhages [12]. As DR progresses, hard exudates occur due to the disruption of the blood–retinal barrier and leakage of inflammatory cytokines and plasma proteins. Further vasoconstriction and capillary occlusions lead to retinal ischemia with the characteristic presence of ‘cotton wool spots’. In the advanced stages of DR, severe hypoxia leads to neovascularization, vitreous hemorrhage and retinal detachment [13]. Given the significant rise in the incidence of T1DM and the increased life expectancy for these patients, the vision impairment associated with DR will unavoidably grow in the upcoming years.

The development of DR is mainly correlated with the duration of diabetes, hyperglycemia and subsequently elevated HbA1c levels. However, despite these well-known risk factors, there is a considerable variation in the development and progression of DR in T1DM patients, which cannot be fully explained by these factors alone. Thus, identifying imaging biomarkers to stratify the risk of developing DR is essential, especially in children with T1DM.

Currently, for the diagnosis and staging of DR, multiple imaging protocols are used, including dilated fundus examination, fundus fluorescein angiography (FFA) and optical coherence tomography (OCT) [14]. FFA has been considered the most valuable method for diagnosing and monitoring DR for many years, providing detailed information about the vascular integrity of the retina. FFA is an invasive test using a contrast technique associated with multiple risks for T1DM patients [15]; therefore, it is rarely used for this category of patients and is not suggested for eyes without visible signs of DR [16].

Over the past few years, non-invasive and fast imaging methods like optical coherence tomography angiography (OCTA) have provided improved visualization of the retinal and choroidal vascular networks without the need for contrast agents. The OCTA technique analyzes the retinal superficial capillary plexus (SCP) and the deep capillary plexus (DCP) separately [17], providing a deeper understanding of the pathophysiological changes in DR; it also provides detailed three-dimensional information about early microcirculatory disturbances [18].

The OCTA system implemented in our current study utilizes a split-spectrum amplitude-decorrelation angiography algorithm. This allows for both qualitative and quantitative evaluation of microvascular alterations within the various layers of the retina, focusing on a 6 mm × 6 mm area centered around the fovea. [19].

Although multiple clinical studies have focused researching correlations between microvascular retinal alterations and clinical parameters in adult type 2 diabetic patients, only a few have researched these possible correlations in young patients with T1DM without signs of DR. The purpose of our current study was to identify the correlations between the retinal microvascular alterations quantifiable on retinal OCTA scans and clinical parameters of young T1DM diabetic patients without clinical signs of DR. 

## 2. Materials and Methods

This retrospective, exploratory enrolled 69 patients aged between 5 years old and 30 years old diagnosed with T1DM and monitored in the Department of Pediatrics of the Clinical Emergency Hospital and ATB Ophthalmology Medical Center, Craiova, Romania. The duration of the study was 2 years, starting from 2021 until 2023. All the study participants were receiving exogenous insulin treatment via insulin pumps or insulin pens. The study protocol was approved by the Ethics Committee of the University of Medicine and Pharmacy Craiova (Project Identification Code 8612/07/06/2021) and was carried out in accordance with the rules of the Declaration of Helsinki, revised in 2013. Written informed consent was obtained from all the patients included in the study and the legal guardians of patients younger than 18 years old.

The study’s inclusion criteria were as follows: patients with T1DM diagnosis based on the International Society for Pediatric and Adolescent Diabetes (ISPAD) guidelines, absence of clinical signs of DR, a minimum disease duration of one year from onset to the time of the study, no other ocular or systemic conditions, except for refractive errors up to 1.5 spherical or cylindrical diopters. T1DM disease duration was determined by medical record review. Exclusion criteria consisted of: patient’s age above 30 years old, inability to stay still while the OCTA scans were taken, history of significant ocular injury or ocular disease, as well as any other systemic pathology that could affect the retinal microvasculature.

All the study participants underwent a comprehensive ophthalmic examination which included: best-corrected visual acuity (BCVA) (LogMAR), intraocular pressure measurement using the IC 100 ICare tonometer, (ICare, Vantaa, Finland), slit-lamp biomicroscopy examination and dilated fundus examination using tropicamide 1% eye drops. Refractive measurements were carried out with the help of a Nidek Ark-1 refractor–keratometer device.

The OCTA scans for the evaluation of the retinal microcirculation were taken with the help of the RevoNX 130 OCTA device (Optopol, Zawiercie, Poland). The scans were performed on dilated eyes using 1% tropicamide eye drops. 

The OCTA software 10.0.1 automatically analyzed the following parameters: foveal avascular zone (FAZ) area, perimeter and circularity, overall foveal thickness, SCP and DCP vessel densities.

FAZ represents the capillary-free area located at the center of macula and surrounded by the foveal capillary circles [20]. The physiological FAZ parameters in healthy subjects are considered to be the following: FAZ area 0.28 ± 0.23 mm^2^ [21], FAZ perimeter 3.3 ± 1.0 mm and FAZ circularity index 0.46 ± 0.1 [22]. The normal shape of the FAZ in healthy subjects is circular or slightly elliptical, with a mean circularity index of 0.8 or more [23]. 

The SCP is located in the nerve fiber and ganglion cell layers, at the same level as the arterioles and major venules, while the DCP is located between the inner nuclear layer and the outer plexiform layer.

Only high-quality scans without blink artifacts, motion artifacts or low signal strength were taken into consideration for statistical interpretation. The scans focused on an area of 6 × 6 mm centered around the fovea, and they were “en face superficial” and “en face deep” reports. In the context of OCTA, the term “en face” refers to a view of the retina that is seen from above. This view contrasts with cross-sectional views typically seen in traditional OCT imaging. While cross-sectional (B-scan) images provide detailed information about the different layers of the retina in a slice-like fashion, “en face” OCT images allow observation of the retinal structures in a planar view, which can be particularly useful for visualizing the layout of retinal blood vessels and microvasculature [24].

We created a customized software which uses OCR technology to automatically extract data from the PDF reports generated by the OCTA software 10.0.1.

In our previous research, we compared the OCTA retinal parameters of this study group with a control group of healthy subjects of the same age group and observed the following statistically significant differences: decreased FAZ area and circularity, increased FAZ perimeter, decreased foveal thickness and vessel density in the SCP for the T1DM group. Further on, for the current study, we chose to explore if there were any specific clinical characteristics of the T1DM group that could be correlated with alterations of the above-mentioned OCTA parameters.

The following clinical parameters were analyzed for this study: the patient’s age at the onset of the disease, the duration of T1DM, BMI at the time of enrollment in the study, the HbA1C values at onset and the mean values of HbA1C over the monitorization to the disease and the degree of DKA at onset.

### Statistical Analysis

The data gathered from the OCTA reports was processed using Microsoft Excel 2019 (San Francisco, CA, USA). For the statistical analysis of the results, Pearson’s and Spearman’s correlation coefficients were used for correlation analysis with *p* < 0.05 considered statistically significant and unpaired *t*-tests were used to compare the study subgroups. The analysis was produced using a commercial software (SPSS version 26.0; IBM, Armonk, NY, USA).

## 3. Results

The present study included 69 patients ranging in age from 5 to 30 years old, with an average age of 12 years and 4 months old. The mean duration of the disease was 5 years old. The study patients began showing symptoms of T1DM at ages ranging from 1 year and 3 months up to 15 years old, with the average age at onset being 7 years and 4 months. There were no statistically significant differences in the age of onset between male and female patients.

### Top of Form

The average weight of the T1DM patients at the time of entering the study was 50.47 kg and the average height was 153 cm, with an average BMI value of 21.4 kg/m^2^. The average HbA1C values at the onset of the disease were 12.4% and the mean HbA1C values when entering the study were 7.4% as described in Table 1.

The metabolic control of the study patients was evaluated with the following parameters: HbA1C at onset and the mean value of HbA1C for the duration of the disease calculated by the average value of 4 measurements per year since onset. The grade of ketosis and ketoacidosis (DKA) at the onset of T1DM was also analyzed with DKA being defined as mild by a pH level of 7.25–7.3 and a serum bicarbonate level between 15–18 mEq/L, moderate DKA when pH levels reach 7.0–7.24 and a serum bicarbonate level of 10 to less than 15 mEq/L and severe DKA when pH levels drop lower than 7.0 and bicarbonate levels lower than 10 mEq/L.

At the onset of the disease, the percentage of patients with ketosis was 13.4%, mild DKA 11.9%, medium DKA 44.8% and severe DKA 29.9%, as described in Table 2.

Family history of DM was also analyzed and 52.2% of patients had no family history of DM, 37.3% had at least one grandparent with DM, 9% had at least one parent with DM and 1.5% had one brother with DM.

**Figure 1:**
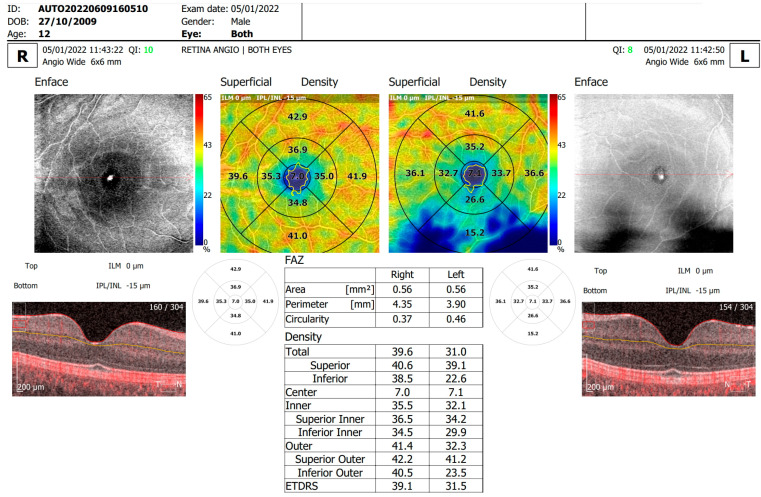
OCTA scan of a T1DM 12-year-old patient with altered FAZ parameters, foveal thickness and vessel densities.

**Figure 2:**
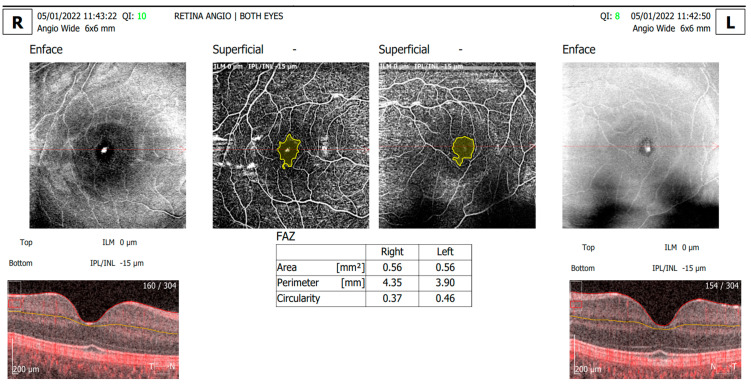
Angio wide OCTA scan of the same T1DM 12-year-old patient showing retinal vasculature of the SCP.

Our assessment focused on the relationships between various OCTA parameters—including the FAZ area, perimeter, circularity, overall foveal thickness, and vessel density in both the SCP and DCP within a 6 × 6 mm area centered on the fovea (as shown in Figure 1 and Figure 2)—and the clinical characteristics of the study patients.

For our study group, the FAZ area and perimeter correlated positively with the mean values of HbA1C (Table 3). There was no significant correlation between the OCTA parameters and T1DM duration and patient’s age at onset.

Regarding the vessel density parameters, the total vessel density in the SCP correlated negatively with the duration of T1DM, based on the superior and inferior analyzed areas as shown in Table 4. The foveal thickness also correlated negatively with the levels of DKA at onset and the levels of HbA1C at onset as shown in Table 5.

Further on, the study patients were distributed in two groups according to the duration of the disease: group 1 with a duration of T1DM of less than 5 years, which included 32 patients and group 2 with a duration of T1DM of more than 5 years, which included 37 patients. Independent unpaired *t*-tests were used to compare the OCTA retinal parameters for the two groups.

**Figure 3:**
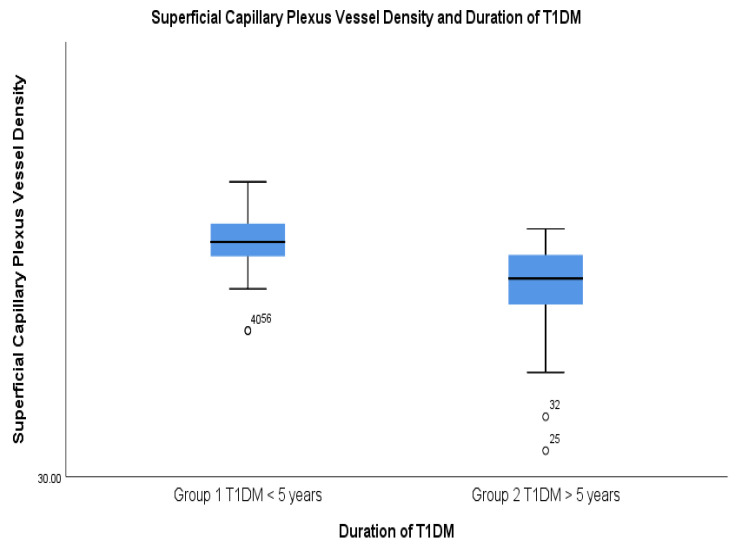
Decreased SCP vessel density in the T1DM with a duration of more than 5 years.

**Figure 4:**
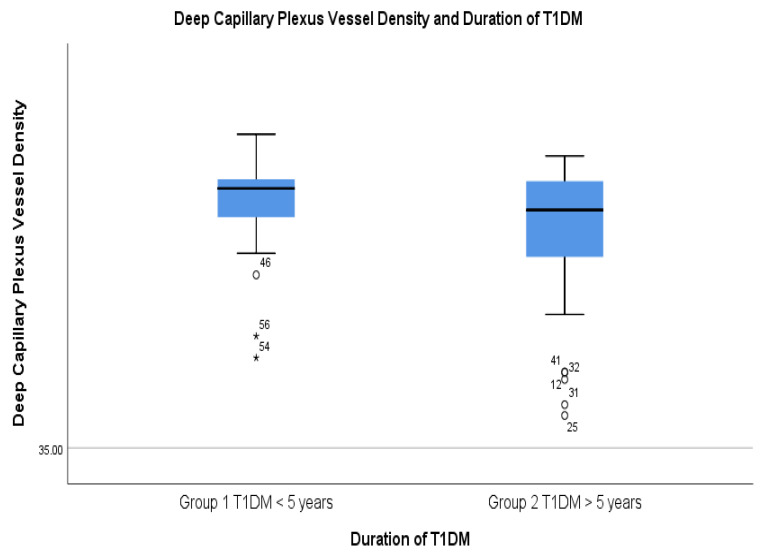
Decreased DCP vessel density in the T1DM with a duration of more than 5 years.

While the FAZ related parameters didn’t show significant statistical differences between the two groups, the vessel densities in both SCP and DCP were significantly lower in group 2, which had a duration of the disease of longer than 5 years (Figure 3 and Figure 4). 

These parameters were located in the SCP in the superior and inferior outer region, as well as the superior and inferior inner region. In the DCP, the statistically significant differences were found in the outer inferior region and deep inner superior region as shown in Table 6.

## 4. Discussion

One of the main risks of T1DM in young patients is the development of microvascular complications at an earlier stage of life [25]. For these patients, the development of DR which is considered a neurodegenerative disease of the retina [26], leads to a higher risk for vision loss as the duration of diabetes increases. However, vision loss due to DR is largely preventable in patients with T1DM, with early diagnosis and appropriate treatment. As FFA is not a routine investigation for patients without visible retinopathy, especially not for young T1DM patients, OCTA represents a valuable noninvasive alternative which can be used to identify retinal microvascular changes in T1DM patients before clinically visible retinopathy occurs [27].

The FAZ is a rod-free area located at the macula, crucial for central vision due to its high density of cone photoreceptors [28]. This zone lacks vasculature and overlying inner retinal tissue, enhancing optical quality by minimizing light scattering [2]. In DR, the FAZ undergoes significant changes, mainly due to the loss of capillaries in nearby vessels. The mechanisms behind capillary loss are based on chronic hyperglycemia-induced damage, such as: pericyte loss affecting the capillary walls, microvascular occlusions, endothelial dysfunction, and breakdown of the blood-retinal barrier [29]. Capillary loss also leads to decreased vessel density in the SCP and DCP, promoting local ischemia and subsequent increase in vascular endothelial growth factor. Therefore, examining FAZ parameters and retinal capillary vessel densities can provide comprehensive understanding of the systemic microvascular alterations occurring in diabetic patients.

In recent years, OCTA retinal screening is preferred to FFA, due to its noninvasive character [30], but also for the detailed imaging properties. Jung JJ et al. [31] demonstrated that retinal OCTA imaging improves the visualization of retinal capillaries and increases the clarity of the FAZ borders compared to FFA, therefore emphasizing the correlation between retinal vessel density and FAZ-specific parameters to DR severity. Moreover, OCTA has been proven to provide valuable information about early changes of parafoveal capillary plexuses in patients without clinical signs of DR [32].

Until now, significant correlations between the OCTA microvascular retinal changes and clinical parameters have not been sufficiently researched in T1DM young patients without DR. The results of our current study focused in two directions. Firstly, the changes in FAZ area and perimeter were found to correlate positively with the HbA1C mean value. Although the FAZ related parameters did not correlate with the duration of the disease, the SCP total vessel density correlated negatively with the duration of T1DM.

From the perspective of the FAZ changes, our results are in line with previous clinical studies such as Wysocka-Mincewicz M et al. [33] who found that higher levels of HbA1c corresponded to a larger FAZ area and decrease in foveal vessel density. Contradictory to our results, in their study on OCTA vessel density in children with type 1 diabetes Gołębiewska J et al. [34] did not find statistically significant correlations between FAZ area or vessel densities of the SCP and DCP and the duration of the disease, age of onset or mean levels of HbA1C.

Also, previous research conducted by Marwa Abdelshafy & Ahmed Abdelshafy [35] found significant correlations between FAZ area and perimeter and T1DM duration.

Regarding the study of vessel density perfusion in the SCP, Jing Qian et al. [36] found that the perfusion density was significantly decreased as the duration of DM increased.

Early detection of key clinical characteristics of the T1DM patients which lead to preclinical microvascular retinal alterations identifiable with OCTA is essential, as DR is one of the pathologies benefiting the most from significant advances in artificial intelligence (AI) analysis. Deep learning-based OCTA image analysis has currently become accurate and efficient regarding image quality control, segmentation, and classification, leading towards probable changes in the DR staging system in the near future [37]. A consensus regarding the correlations between OCTA retinal imaging biomarkers and clinical parameters is essential and can facilitate the routine clinical use of OCTA for early detection and management of DR for young T1DM patients.

The current study presents several limitations, such as: the current macular OCTA protocol has a field of view of 6 × 6 mm centered on the fovea, therefore the peripheral vascular layers were not evaluated. The accuracy of OCTA measurements is crucial for proper interpretation of the results. However, algorithms in OCTA machines, like any other automated system, can occasionally produce incorrect measurements due to various factors such as image quality, motion artifacts, or segmentation errors. The OCTA device that we used for the current study comes with a software that allows for some degree of manual correction or editing of the retinal parameters. We aimed not to use these editing and correction features, but rather to repeat the scans until proper quality and minimal motion artifacts were obtained.

The final number of participants included in the study is relatively small because of the reluctance of some of the patient’s parents for consenting to be part of scientific research on one hand, on the other hand some T1DM patients also presented other ocular or systemic pathologies and we also encountered difficulties in obtaining high-quality images from children younger than 5 years old. As the duration of the disease is considered one of the critical risk factors for the progression towards DR, we decided to divide the T1DM group into 2 subgroups based on this parameter, where group 1 had a duration of the disease of up to 5 years and group 2 had a duration of more than 5 years. Ideally, a third group with a duration of T1DM of more than 10 years would have added more statistical value to the research, but out of the 69 study patients only 7 of them had a duration of the disease of more than 10 years, therefore a valid comparison with the other groups could not be made. We started performing the OCTA scans for the T1DM patients respecting the protocol for retinal screening, which advises a duration of at least one year since the diagnosis of the disease, but at the time we started the study most patients already had a duration of more than 1 year since the onset of T1DM. The patients attended only one visit for the OCTA assessment, meaning that, for now we have no follow up scans to enable us to further evaluate the progression of the OCTA retinal alterations. We aim for further research of this study group in order to obtain OCTA reports of progression for these patients and gain dynamic insights on their clinical parameters as the duration of the disease increases.

## 5. Conclusions

OCTA retinal scanning represents a reliable, noninvasive method to detect early retinal microvascular alterations in young T1DM patients without clinical signs of DR. These microvascular changes can be regarded as specific imaging biomarkers and are correlated with various clinical parameters such as: the FAZ area and perimeter increased with higher mean values of HbA1C, therefore with a poor metabolic control. Moreover, the SCP total vessel density decreases as the duration of T1DM increases. Regarding the vessel densities in the SCP and the DCP, we found that they decrease with a duration of the T1DM of more than 5 years.

Early detection of retinal microcirculatory alterations with the help of OCTA as well as proper management of the controllable clinical factors which lead to these alterations may delay the clinical diagnosis of DR and orientate towards a different approach in the overall clinical management of T1D patients.

OCTA is becoming an essential tool for monitoring the progression towards clinical signs of DR in young T1DM patients. While we can not control some clinical factors which lead to these alterations, like the duration of the disease, we can improve the metabolic control and raise awareness regarding the onset clinical signs and diagnose children with T1DM before severe DKA occurs.

## Figures and Tables

**Table 1:** Clinical characteristics of the study patients.

	Minimum	Maximum	Mean
Age (years)	4	20	12.4
Duration of T1DM (years)	1.50	23.83	5.04
Age onset (years)	1.25	15.16	7.40
Height (cm)	100	176	153.10
Weight (Kg)	15	115	50.47
BMI (kg/m^2^)	12.57	37.55	21.40
HbA1C onset (%)	7.54	18.60	12.41
HbA1C Mean Value (%)	5.20	13.90	7.43

**Table 2:** Degree of DKA at T1DM onset.

	Frequency	Percent	Valid Percent
Mild DKA	30	43.5	43.5
Moderate DKA	20	29.0	29.0
Severe DKA	10	14.5	14.5
Ketosis	9	13.0	13.0
Total	69	100	100

**Table 3:** Correlations between FAZ Parameters and HbA1C Mean Values.

FAZ Parameter	HbA1C		
	Pearson Correlation	Sig. (2-Tailed)	N
Area	0.246 *	0.044	68
Circularity	−0.093	0.449	68
Perimeter	0.248 *	0.041	68

* Correlation is significant at the 0.05 level (2-Tailed).

**Table 4:** Spearman correlation between Vessel Density Parameters and Duration of T1DM.

	Duration of T1DM		
	Spearman Correlation Coefficient	Sig. (2-Tailed)	N
SCP Region Total	−0.365 **	0.002	69
SCP Region Superior	−0.272 *	0.024	69
SCP Region Inferior	−0.237 *	0.050	69

* Correlation is significant at the 0.05 level (2-Tailed). ** Correlation is significant at the 0.01 level (2-Tailed).

**Table 5:** Spearman correlation between Foveal Thickness and metabolic parameters at onset of T1DM.

	Foveal Thickness		
	Spearman Correlation Coefficient	Sig. (2-Tailed)	N
DKA at onset	−0.256 *	0.034	69
HbA1C at onset	−0.240 *	0.047	69

* Correlation is significant at the 0.05 level (2-Tailed).

**Table 6:** Unpaired T Test Descriptive for the 2 groups.

OCTA Parameter	Group 1/Group 2	Mean	Std. Deviation	Std. Error Mean
Area	1	0.3822	0.12795	0.02262
	2	0.3984	0.12751	0.02096
Circularity	1	0.4466	0.11418	0.02018
	2	0.4368	0.11003	0.01809
Perimeter	1	3.3303	0.80315	0.14198
	2	3.4351	0.79128	0.13009
SCP Total Region	1	38.8438 *	1.24303	0.21974
	2	37.0919 *	1.96601	0.32321
SCP Superior Region	1	39.2813 *	1.78370	0.31532
	2	37.6676 *	1.90876	0.31380
SCP Inferior Region	1	38.2281 *	1.71167	0.30258
	2	36.3973 *	3.40412	0.55963
SCP Center Region	1	14.8031	5.32556	0.94144
	2	12.8405	4.93674	0.81159
SCP Inner Region	1	37.2438 *	2.83150	0.50054
	2	35.1703 *	2.87797	0.47314
SCP Superior Inner Region	1	37.9688 *	2.95410	0.52222
	2	35.6243 *	3.52620	0.57970
SCP Inferior Inner Region	1	36.4469 *	3.06257	0.54139
	2	34.6703 *	3.08643	0.50741
DCP Total Region	1	41.7906 *	1.36957	0.24211
	2	40.8838 *	2.00562	0.32972
DCP Superior Region	1	41.8406	1.85383	0.32771
	2	41.2081	2.11389	0.34752
DCP Inferior Region	1	41.6188 *	1.41066	0.24937
	2	40.5108 *	2.95642	0.48603
DCP Center Region	1	29.6438	4.67808	0.82698
	2	30.0757	4.77641	0.78524
DCP Inner Region	1	43.3969 *	0.99336	0.17560
	2	42.6703 *	1.37313	0.22574
DCP Superior Inner Region	1	43.5750 *	1.05678	0.18681
	2	42.7784 *	1.57218	0.25846
DCP Inferior Inner Region	1	43.1969	1.18580	0.20962
	2	42.5622	1.59659	0.26248
Foveal thickness	1	224.50	15.100	2.669
	2	220.30	18.625	3.062

* Significant statistical difference between Group 1 and Group 2.

## Data Availability

The authors declare that the data for this research are available from the correspondence authors upon reasonable request.
